# The impact of multiplex panel testing on ascertainment of pertussis-attributable, non-infant deaths: national surveillance from England

**DOI:** 10.1128/jcm.01856-25

**Published:** 2026-07-10

**Authors:** Catherine Sandford Alber, Emer Cullen, Sonia Ribeiro, David J. Litt, Gayatri Amirthalingam, Helen Campbell, Sharif A. Ismail

**Affiliations:** 1 South East Health Protection Team, UKHSA 371011, London, United Kingdom; 2 North East Health Protection Team, UKHSA 371011, London, United Kingdom; 3 Immunisation and Vaccine Preventable Diseases Division, UKHSA 371011, London, United Kingdom; 4 Respiratory and Vaccine Preventable Bacteria Reference Unit, UKHSA 371011, London, United Kingdom; 5 Immunisation Programmes Division, UKHSA 371011, London, United Kingdom; Maine Medical Center Department of Medicine, Portland, Maine, USA

**Keywords:** pertussis, whooping cough, mortality, surveillance, multiplex panel testing, multi-pathogen testing

## Abstract

Understanding the burden of pertussis and pertussis-related mortality is important in enabling a better understanding of population vulnerability and supporting vaccination program planning. However, changes in laboratory practice can have important implications for public health surveillance. This study considers the impact of increasing use of multiplex panel testing for respiratory pathogens on the ascertainment of pertussis-related non-infant deaths, drawing on multiple surveillance data sources from England. Information was sourced from the Office of National Statistics cause of death records, public health case management records, laboratory results collated nationally, and death notifications received via General Practices. Figures covered the period 2023/24, coinciding with a large, national outbreak in England, and were compared with data from 2012—the last major outbreak in England. Of 34 deaths identified as potentially pertussis-related among individuals aged >12 months in 2023/24, 11 ultimately met the pre-defined criteria to be classified as pertussis-attributable; none of the five possible cases identified in 2012 did so. A total of 29 of the 34 (85%) tested positive for pertussis via multiplex panel testing, of which 17 out of the 29 (59%) were ultimately excluded as pertussis-related deaths. Findings highlight significant complexity in the attribution of pertussis-related mortality in an era of widespread multiplex panel testing.

**IMPORTANCE:**

Correctly identifying deaths caused by pertussis (whooping cough) is important for timely public health action but is not straightforward. We aimed to find out how many deaths potentially caused by pertussis that are notified to national authorities in England are really caused by the infection, in a context where many hospitalized patients are now being tested using multiplex panel testing. Overall, 68% of those cases identified as potential pertussis deaths through regular pertussis surveillance processes in England proved not to have been caused by the infection. This finding highlights challenges to surveillance of pertussis-related deaths in the era of widespread multiplex panel testing use. It may point to the need for clearer guidance concerning the use of this testing approach.

## INTRODUCTION

Pertussis is endemic worldwide, and despite the implementation of long-standing vaccination programs, it remains a cyclical disease with peaks in cases occurring every 3–5 years globally (1, 2). The primary objective of the pertussis immunization program in England is to prevent deaths in infants, the highest risk group for severe outcomes. There are more limited data internationally on the risk of pertussis in older children, adolescents, and adults, and reliably assessing the true morbidity and mortality attributable to pertussis is more challenging.

Measures introduced during the COVID-19 pandemic resulted in very low rates of pertussis in 2021 and 2022 in England (3) and indeed across Europe (4). In England, there was a surge in cases across all age groups from summer 2023 (5) continuing into 2024, with 14,894 laboratory-confirmed cases in 2024 (6). This was the largest outbreak in England since 2012, which, at the time, had been the largest increase in pertussis cases in over 20 years (7). The 2023/24 outbreak was also the largest in England since the introduction of the maternal vaccination program, which commenced in October 2012 (7). A similar increase was seen in some countries across Europe, with notification rates in the European Union ranging up to 77.53 per 100,000 (2, 8).

Routine surveillance for pertussis cases is carried out across Europe to monitor circulating pertussis levels, and several European countries routinely report on pertussis-related deaths. The European Centre for Disease Prevention and Control’s annual pertussis report for 2023 documents 10 deaths across the continent affecting infants and those in older age groups (8). In England, infant deaths from pertussis are routinely reported, and data on these deaths form an important part of ongoing work to monitor the effectiveness of maternal and infant pertussis vaccination programs (5). There were 14 infant deaths with laboratory-confirmed pertussis reported in 2012 and 11 in 2024 (9).

The process for attribution of deaths due to pertussis is complex. In England, a key source is death certification from the Office for National Statistics (ONS), but in some cases, final judgment on the cause of death may be delayed (e.g., where a post-mortem or case investigation may be required). Laboratory testing is central, but there have been important changes in many countries in testing approaches prior to, and since the pandemic, which influence mortality attribution.

The most common laboratory techniques for detecting *Bordetella pertussis* infections are culture, polymerase chain reaction (PCR), and immunological assays (e.g., enzyme-linked immunosorbent assay [ELISA] and multiplexed immunoassays [MIA]) for serological testing (10, 11). Testing approaches vary by age group—PCR being the mainstay for case confirmation in infants and younger age groups. PCR detection of *B. pertussis* in respiratory samples is recommended during the first 3–4 weeks of symptoms; tests may incorporate multi-copy gene targets such as IS*481* (for maximum sensitivity, with a small compromise on specificity) and/or single-copy targets such as *ptxP* (for optimal specificity, at the expense of sensitivity) (10). However, multiplex PCR panels are now widely used in England and in many other high-income countries worldwide (12). Multiplex panel testing involves the simultaneous testing of a single sample for multiple pathogens using PCR. *B. pertussis* PCR is included in a range of respiratory multiplex PCR panels alongside common respiratory viruses such as influenza, RSV, and SARS-CoV-2. The use of multiplex panels can reduce the sensitivity for pertussis detection (13, 14) but may also result in the detection of cases that would not have otherwise been tested for pertussis due to atypical presentation or lack of clinical identification, the latter being more likely to occur in non-infant age groups.

The analysis reported in this paper aimed to assess the impact of the increasing use of multiplex panel testing on pertussis-related death ascertainment. We analyzed data on non-infant pertussis deaths in England from the 2023/24 outbreak and compared the findings with data from the last major outbreak in 2012—a period when multiplex panel testing was not available.

## MATERIALS AND METHODS

### Data sources

The UK Health Security Agency (UKHSA) collects routine surveillance on any deaths, which may be related to *B. pertussis* infection. Non-infant pertussis-related death data are collated from the following sources:

- Notified cases (regardless of laboratory status) and cases with laboratory confirmation are collated by regional health protection teams and recorded on the national Case and Incident Management System (CIMS) on an individual level (formerly HPZone until summer 2024, and then transitioned to CIMS). These are reviewed for information indicating a death.
- Deaths with pertussis as an underlying cause recorded by the ONS in cause of death (COD) records.
- Laboratory-confirmed cases of pertussis where a death is recorded in the NHS Personal Demographic Service in temporal association with the laboratory result. These laboratory results may be from the national reference laboratory at Colindale, UKHSA regional laboratories via Data Mart or from local NHS laboratories with results recorded centrally on the second-generation surveillance system (SGSS), and the national laboratory reporting system used in England to collect data on infectious diseases.
- Death notification via the enhanced surveillance form that is disseminated to the General Practitioners (GPs) with whom each laboratory-confirmed case is registered.

The collated list of possible pertussis deaths for 2012 and from January 2023 to December 2024 was reviewed to compare cases who had died from the two most recent outbreaks. Where the cause of death was not reported in ONS or laboratory results were unavailable, further information was obtained from review of the CIMS/HPZone record and, if required, by contacting the registered GP, the trust-based microbiologist, or local coroner’s office. For cases aged under 18 years of age, additional information was also obtained from the National Childhood Mortality Database (NCMD).

### Outcome definitions

Pertussis-related deaths were defined in this analysis as those in individuals aged over 12 months of age with either of the following.

#### Primary definition

Pertussis is listed in the definitive COD from the ONS as part of the illness episode leading to death, and a positive pertussis laboratory test result was recorded up to a maximum of 12 weeks prior to the date of death, or where this information was not available.

#### Secondary definitions

Either (i) a positive pertussis laboratory test result with clinically compatible symptoms or, in the absence of a test result, (iia) clinically compatible symptoms and no more likely diagnosis or (iib) clinically compatible symptoms and equivalently possible diagnosis, and also a pertussis epidemiological link per case definition. Clinically compatible symptoms were defined as per the case definition in the UKHSA Pertussis Management Guidance for England (15).

### Further investigation and analysis

For cases occurring in 2023/24 with a positive pertussis test result, the relevant hospital laboratory was contacted to determine the type of testing used, and the clinical information was provided with the request for pertussis testing. If applicable, laboratories were asked for the type of multiplex panel testing used and checked for UKAS accreditation. Cases with a positive test result from 2012 were assumed to not have used multiplex panel testing, as they occurred prior to the introduction of multiplex panel testing in hospital laboratories. Data from all sources above were collated and analyzed descriptively in MS Excel.

## RESULTS

In 2012, of the five non-infant deaths identified through routine surveillance routes as possible *B. pertussis*-related deaths, none met the primary definition of pertussis-attributable deaths applied in this study (i.e., pertussis was not listed as a cause of death). The deaths were detected through laboratory reporting (3) and GP notifications (2), which are non-infant deaths with laboratory-confirmed pertussis near the time of death. The laboratory confirmation method for all the cases was via serology. The median age was 69 years (all non-infant deaths were in individuals in the age range between 55 and 75 years; Table 1).

**TABLE 1:** Age range for all notified individuals in 2012 and 2023/24, in the 10-year age bands

Age range (yrs)	Number of individuals notified in 2012	Number of individuals notified in 2023/24	Number of individuals with confirmed pertussis-related death in 2023/24
1–9	0	2	0
10–19	0	1	0
20–29	0	0	0
30–39	0	1	1
40–49	0	1	0
50–59	1	2	0
60–69	2	8	4
70–79	2	9	1
80–89	0	9	5
90–99	0	1	0

**Fig 1:**
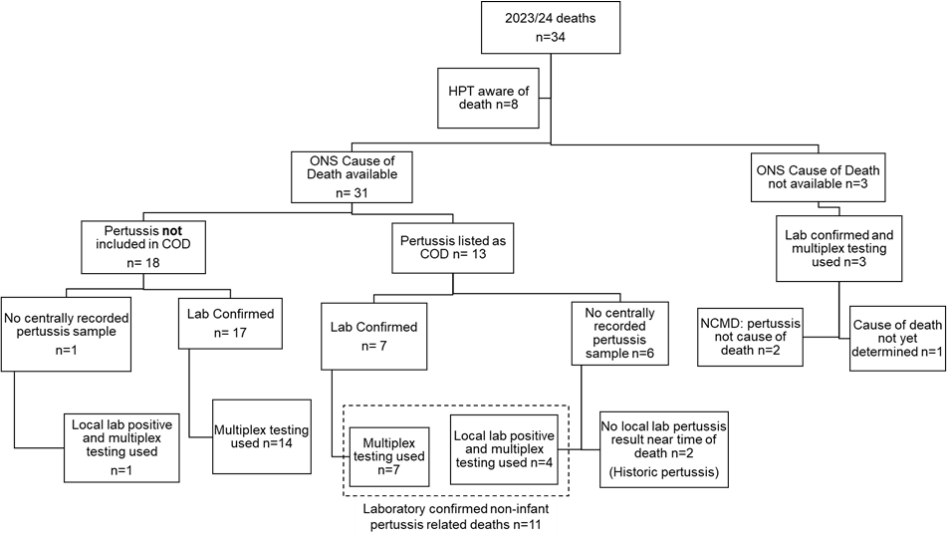
Flow-chart for notified possible pertussis-related deaths in England 2023/24, with an ensuring breakdown outlining numbers laboratory-confirmed, and those testing positive specifically via multiplex panel testing. [Abbreviations: COD = Cause of death; HPT = Health Protection Team; ONS = Office of National Statistics]

In 2023/24, there were 34 non-infant deaths identified as possible pertussis-related deaths. Nineteen were reported through pertussis database laboratory results, one from GP, seven from ONS death records, and seven from CIMS/HPZone (Fig. 1). The median age was 75 years (see Table 1 for age range of individuals). From the deaths identified, 31 (91%) had an ONS cause of death available. Of these, 13 (38%) met the primary definition of pertussis being listed as a contributory cause of death, of which two deaths in adults were excluded because primary infection occurred many years previously, in childhood. All three cases where the ONS cause of death was unavailable were 1–19 years of age. For two of these cases, information was obtained from the NCMD, and the Child Death Overview Panel (CDOP) determined that pertussis was not a contributory factor to the cause of death. For the remaining cases, the cause of death had not yet been determined at the time of this paper’s publication. This gave a total of 11 laboratory-confirmed pertussis-related non-infant deaths in 2023/24. In these 11 deaths, the median age was 75 years (all deaths were in individuals in the age range between 30 and 39 and 60 and 89 years). All the cases were tested pertussis-positive via multiplex panel assays, and none of the cases had pertussis listed as the only cause of death. Most cases had at least one other chronic condition listed, with chronic respiratory illness being listed for 4 out of the 11 cases. Six of the cases had an additional respiratory pathogen listed.

Of the 34 cases notified in 2023/24, 32 were found to have laboratory-confirmed pertussis, with five having no centrally recorded result on SGSS, but a confirmed positive test in the local laboratory on further enquiry. Of the 32 cases with laboratory-confirmed pertussis, multiplex panel testing had been used in 29 (91%) to identify pertussis. Out of these 29 cases, 11 (38%) met the primary definition of a pertussis-related death, 17 (59%) did not have pertussis included in the ONS cause of death or the CDOP decision, and one had a cause of death still to be determined.

In total, 17 hospital microbiology laboratories were contacted (covering all those laboratories from which one or more positive pertussis results were recorded), 11 of which had UKAS accreditation for multiplex panel testing for *Bordetella pertussis*. Some of the hospital laboratories received samples from other hospitals and acted as the central site offering multiplex panel testing. A range of platforms were used, including Qiagen Qiastat, Biomerieux Biofire, and Aus Diagnostics. The criteria used for multiplex panel testing varied across the country and included immunocompromise, high dependency or intensive care unit admission, pediatric admission, severe illness, and an urgent test request. Many laboratories required microbiology consultant approval to use multiplex panel testing, but one laboratory routinely tested all throat swabs.

## DISCUSSION

Understanding the true incidence of pertussis and pertussis-related mortality can help us to highlight the areas of unmet need or vulnerable populations and support vaccination planning (16). Changes in detection and case management approaches between the 2012 and the 2023/2024 outbreak mean that comparison of the number of non-infant deaths in the two time periods should be undertaken with caution. There were 8,859 laboratory-confirmed pertussis cases in England in this age group in 2012, compared to 13,986 in 2024 (9). In the 2012 outbreak in England, we identified no non-infant deaths meeting the pertussis-related death definition applied in this analysis, compared to 11 deaths in 2023/24 that did meet the definition. Importantly, of 34 deaths potentially attributable to pertussis in 2023/24, 29 (85%) tested positive via multiplex panel testing, of which 17 out of the 29 (59%) were ultimately excluded as pertussis-related deaths according to the primary definitions used in this analysis, with one case still awaiting a definitive decision on cause of death. This finding suggests that a substantial proportion of those originally notified as potential pertussis-related deaths in 2023/24 may have had evidence of *B. pertussis* carriage without disease.

Nearly half (45%) of the pertussis-related deaths in England in 2023/24 were in individuals aged over 80 years of age, and the majority (91%) were aged over 60 years (Table 1). All cases had more than one cause of death listed in addition to pertussis. Most cases had another chronic illness, with chronic respiratory illness being the most common additional cause of death. However, given the small numbers and the fact that these deaths occurred during a period of very high transmission in England with exceptionally high case numbers reported overall, these numbers should be interpreted with caution.

While it is not the purpose of this analysis to evaluate the performance of different multiplex panel testing assays, findings from this analysis underscore the complexity in attribution of pertussis-related mortality in an era of widespread, extended multiplex panel testing, including for pertussis. Multiplex panel testing is now widely used in hospital laboratories in England. The majority of trust laboratories reported using multiplex only on patients meeting certain criteria, such as those who are immunosuppressed, on intensive care or high dependency, having severe illness, or requiring urgent test results. Only a small minority routinely tested all respiratory swabs using a multiplex panel. While international data are sparse, studies suggest that up to one-third of pediatric patients presenting with respiratory symptoms are tested in this way in some settings (17, 18). Studies have also shown that some multiplex PCRs show only 56%–67% positive pertussis rates compared to those identified by single pertussis PCR testing in a cohort of clinically confirmed pertussis patients (10, 13, 14, 19, 20).

The use of multiplex panel testing increases the number of pertussis tests carried out, and this may have led to an increase in case detection and reporting in the 2023/24 outbreak (12, 21). In addition to the use of multiplex panel testing, active case finding, and higher clinical awareness in the 2023/4 outbreak may also have contributed to the higher rates of pertussis-related death detection. It is also notable that the criteria used by many of the hospitals for multiplex panel testing, described above, may make the detection of pertussis more likely in individuals with other medical conditions or comorbidities.

While 55% of the pertussis-related deaths also had another respiratory pathogen listed as a cause of death, it is difficult to determine from this analysis how far each individual infection (or co-infection with multiple pathogens as a distinct risk factor in itself) contributed to death in each case. The use of respiratory multiplex panel testing could increase the likelihood of incidental-positive results. When multiplex panel testing identifies more than one positive organism, it may be difficult to determine whether the infections identified co-contributed to the death or were an incidental finding, depending on the clinical presentation. This is especially the case, given uncertainty concerning asymptomatic, population carriage rates for *B. pertussis*: the (few) available studies assessing this indicate ranges from perhaps 1% to 5% in the general population (22, 23), rising to 50% or more among asymptomatic household contacts of confirmed cases (24), although recent evidence from human challenge studies suggests the potential for higher general population carriage provided sufficient infection pressure (25).

Our analysis also points to broader challenges in death surveillance. In cases without an available ONS cause of death or a laboratory-confirmed pertussis result, obtaining accurate clinical information was sometimes challenging. Several GPs mentioned that they do not receive any information from the coroners regarding the outcome of cause of death decisions, and final ONS cause of death registration can take significant time. In addition, several deaths listed by the ONS with pertussis as a cause of death were found to have no central positive pertussis test result recorded in SGSS. On further investigation, the majority (excluding two historic pertussis cases) did have a positive pertussis result locally, highlighting a potential issue in the transfer of data from local laboratories to SGSS. This may in part be due to increased use of PCR testing, with fewer cultures being sent to the reference laboratory. It requires further investigation to ensure that notifications and potential public health actions are not being missed.

It is possible that the case definitions applied in this analysis may have resulted in true, pertussis-attributable deaths being discounted where the primary case definition was not met. For example, the exclusion of wider diagnostic categories sometimes listed on death certificates (e.g., “Pneumonia, unspecified organism” or “Pneumonia” with no responsible organism indicated) may have resulted in under-counting of deaths. However, including a wider set of diagnostic categories in the primary definition in this way would have precluded pragmatic analysis, given the number of annual reported deaths from pneumonia (all cases) in England (over 18,000 in 2020 [26]).

This analysis highlights complexity in attribution of pertussis-related mortality in an era of widespread multiplex panel testing. The most recent pertussis outbreak (2023/24) saw a higher number of laboratory-confirmed cases of pertussis among non-infants and a higher number of pertussis-related deaths in these age groups than in 2012. This may be the result of changes in pertussis testing and surveillance, including the widespread introduction of multiplex PCR testing, alongside the larger surge in cases in England following the COVID-19 pandemic (2).

## Data Availability

The data that support the findings of this study have been assessed by the UK Health Security Agency Office for Data Acquisition and Release as having sensitive personal information, whose publication may breach the ISB1523: Anonymization standard for publishing health and social care data. Therefore, to protect participants’ privacy, we cannot make this data publicly available. However, some summaries of the data may be available upon reasonable request from the UKHSA. Requests can be directed to DataAccess@ukhsa.gov.uk.
